# Modulation of Glycoprotein VI and Its Downstream Signaling Pathways as an Antiplatelet Target

**DOI:** 10.3390/ijms23179882

**Published:** 2022-08-31

**Authors:** Eduardo Fuentes

**Affiliations:** Thrombosis Research Center, Medical Technology School, Department of Clinical Biochemistry and Immunohaematology, Faculty of Health Sciences, Universidad de Talca, Talca 3480094, Chile; edfuentes@utalca.cl

**Keywords:** glycoprotein VI, platelet, thrombosis, bleeding, pathways

## Abstract

Antiplatelet therapy aims to reduce the risk of thrombotic events while maintaining hemostasis. A promising current approach is the inhibition of platelet glycoprotein GPVI-mediated adhesion pathways; pathways that do not involve coagulation. GPVI is a signaling receptor integral for collagen-induced platelet activation and participates in the thrombus consolidation process, being a suitable target for thrombosis prevention. Considering this, the blocking or antibody-mediated depletion of GPVI is a promising antiplatelet therapy for the effective and safe treatment of thrombotic diseases without a significant risk of bleeding and impaired hemostatic plug formation. This review describes the current knowledge concerning pharmaceutical approaches to platelet GPVI modulation and its downstream signaling pathways in this context.

## 1. Introduction

Atherosclerosis is one of the leading causes of peripheral arterial disease (PAD) and coronary artery disease (CAD) [1]. In this pathological process, the adhesion and aggregation of the platelets following the disruption of the vascular surface is a critical point in thrombus formation [2]. Considering this, the presence of an unregulated platelet activation leads to thrombus formation and possible organ failure [3]. In this sense, antiplatelet therapy is designed to decrease the chance of thrombotic events, but without secondary effects such as a high risk of bleeding [4]. This risk increment is increased in older adults over 75 years old, which can be observed in therapies based on aspirin, prasugrel, and clopidogrel plus aspirin) [5,6,7]. Even the administration of aspirin in a lower dose (primary prevention strategy, 100 mg of enteric-coated aspirin) resulted in a significant increase in the risk of major bleeding with a low therapeutic effect [8]. This problem has led to the need to improve the efficacy of the current approaches, which is the inhibition of the platelet glycoprotein GPVI and associated pathways, a promising therapy without secondary effects [9,10].

Platelet membranes and their glycoproteins offer various receptors to regulate platelet responsiveness under diverse pathophysiological conditions [11].

In particular, the receptor GPVI, a platelet-specific collagen membrane glycoprotein expressed in platelets and megakaryocytes [11], plays a critical role in the arterial thrombosis process because of its associated function with the initial interaction with collagen in the atherosclerotic plaque rupture [12,13,14,15]. Specifically, GPVI initiates the pathway induced by collagen [16,17] through a rapid formation or rise of GPVI dimers, along with vascular injury [18]. This look, reflected in the evidence of higher surface expression levels and the altered activation state of GPVI, has been described in patients with an increased risk of thromboses, such as obese patients [19] and ST elevation myocardial infarction patients [20].

Current evidence points to GPVI as a suitable target in thrombosis prevention with no or only a mild bleeding tendency [21]. This is supported because GPVI is not critical for hemostasis, since the genetic deficiency of GPVI or its dysfunction diminishes the platelet responses to collagen without a bleeding tendency [22,23,24]. Even a total reduction in GPVI improves outcomes in the case of stroke without an increase in cerebral hemorrhage risk [25]. This evidence demonstrates that GPVI is a promising antiplatelet target with minimal effects on hemostasis in pathologies such as thrombosis [20,26,27]. In this context, this review describes the current knowledge regarding GPVI modulation and its downstream pathways as an antiplatelet target.

## 2. Glycoprotein VI and Its Function in Hemostasis

The chromosome responsible for the expression of the human GPVI was mapped to chromosome 19 in the long arm, region one, band three (19q13), along with several members of the Ig superfamily [14]. GPVI is the central platelet receptor for collagen and has been well-described as a 62-kDa glycoprotein located in the membrane and expressed in a noncovalent association with the Fc receptor gamma (FcRγ) chain from murine and human platelets [28,29]. Fcrγ and GPVI form the collagen receptor in platelets, and the activation of this receptor leads to the phosphorylation of FcRγ [30]. The coupling to FcRγ mediated by GPVI is necessary for signal transduction. It is facilitated by an arginine located in the transmembrane region (close to the extracellular side) of GPVI and the intracellular C-tail on the platelet surface [31]. On the other hand, the integrin α2β1 is thought to be essential for platelet adhesion to subendothelial collagen, facilitating subsequent interactions with the activating GPVI [32].

GPVI is initially expressed as a monomer and, through a diffusion mechanism, can form dimers in the membrane, forming a mixture of monomers and dimers on the cell surface [33]. On the platelet, the dimeric form of GPVI shows the highest affinity to fibrous collagen [34,35,36], and this is due to the fact that the association of the GPVI monomer is too low to support a strong binding, which is necessary for the dimerization of GPVI for correct interaction with collagen [37,38]. In dimerization processes, the collagen adhesion induces the clustering of the GPVI dimer, increasing both avidities for collagen and the proximity of molecules in the GPVI-associated signaling [39]. In concordance with the above, the density of the GPVI receptor on the platelet surface is directly proportional to the response to collagen and platelet adhesion [40]. Recently, a structural analysis revealed that GPVI presents a collagen-binding site across the β-sheet of the D1 domain, which are the amino-acids Trp76, Arg38, and Glu40, essential residues for binding to fibrillar collagens and collagen-related peptides (CRPs) [41]. On the other hand, GPVI binds a site on collagen comprising of two collagen chains, with the core formed by the sequence motif OGPOGP. Likewise, this study confirms that GPVI binds sites in collagen created by two of the three triple-helix chains and canonical OGPOGP sequence motifs [41].

Dimeric GPVI can also bind to the fibrinogen D-domain, enhancing the platelet adhesion, activation, and aggregation on immobilized fibrinogen and polymerized fibrin [42,43]. In physiological conditions, a small activation of adhering platelets has been observed because of the high content of plasma proteins in the fibrin formed in blood and plasma that shield against epitopes that could activate GPVI [44]. During the coagulation phase, the monomeric GPVI can cluster fibrinogen through αC-region binding, facilitating the polymerization of fibrin into fibers and thrombin production; however, the dimeric form of GPVI lacks this feature [45,46,47,48,49]. Moreover, the immobilized D-dimer (a fibrin degradation product) can induce platelet spreading. This effect cannot be replicated by the E fragment of fibrin, pointing out these results where only the D-dimer can bind to monomeric GPVI [47]. Likewise, the evidence demonstrates that GPVI can weaken the luminal surface of plaques through a mechanism dependent on fibronectin, enhancing the progression of atherosclerotic disease towards a thrombotic event [50]. Moreover, evidence from the last ten years confirms that GPVI is essential for the repair of neutrophil-induced vascular injury in various inflamed organs and tissues [51,52,53,54].

Structural studies on the interactions between GPVI and different ligands and the related cellular mechanisms of platelet activation have defined GPVI as one of the new antithrombotic therapeutic targets because of the characteristics above. This knowledge has been crucial to designing different pharmacological approaches for inhibiting GPVI and evaluating secondary effects on platelet functionality and survival. These considerations are essential, because platelet activation and inhibition pathways related to GPVI are not exclusive to this protein. In vitro and in vivo studies are necessary to confirm the findings.

## 3. Platelet Signaling Pathways Related to GPVI Activation

The current evidence demonstrates that GPVI has a central role in thrombosis and a minor role in primary hemostasis [55,56,57,58]. Different agonists have carried out different functionalities and pharmacological studies about the GPVI receptor in platelets (collagen, fibrinogen, snake venom toxins, and charged exogenous ligands), but there are three mainly used ones: convulxin (CVX), reelin, and CRP-XL [26]. CVX, a c-type lectin that comes from the venom of a South-American rattlesnake, can bind up to eight individual GPVI receptors and bridge GPVIs on different platelets. Reelin is a secreted glycoprotein that interacts with GPVI with subnanomolar affinity, inducing platelet activation and aggregation. CRP-XL is a triple-helical peptide that represents the synthetic cross-linked collagen-related peptide and is the more specific and potent activator of GPVI for functional analysis in platelets [59]. In the next section, we describe the main pharmacological approaches related to the inhibition of GPVI signaling pathways associated with platelet activation.

### 3.1. Rho/RhoA Kinase Pathway

The activation of the Rho kinase pathway has been described as essential signaling for platelet activation mediated by GPVI, observing that the activation mediated by convulxin was inhibited significantly in platelets with a deficiency in RhoG [60]. Likewise, platelets deficient in RhoG proteins showed an impaired secretion of dense granules and the incapacity to attract other platelets in the thrombi [61]. Moreover, the inhibition of GPVI mediates the Rho kinase pathway without effects on tail bleeding times [61].

### 3.2. PI3K–Akt Pathway

The PI3K–Akt pathway is a key signaling pathway for GPVI downstream activation induced by collagen [62,63,64,65,66]. Specifically, the isoforms α and β of phosphoinositide 3-kinase (PI3K) play an important role for full platelet Ca^2+^ signaling dependent on GPVI, as well as thrombus formation; however, the isoforms δ are not necessary for GPVI activation [62,63,64]. In murine models, the absence or inhibition of PI3Kα showed a small but significant decrease in the size of the thrombi in a simulation of thrombosis in mesenteric arteries, without a modification in bleeding times [65,66]. The inhibition of PI3Kβ by the selective inhibitor TGX221 (2.5 mg/kg IV) in a Folts-like carotid artery stenosis model of thrombosis regulated the blood flow rate to normal values without changing the heart rate, blood pressure, or bleeding time [67]. Moreover, the use of another selective inhibitor of this same protein, called AZD6482, in an in vivo model was associated with a significant antithrombotic effect without an increase in the bleeding times [68]. Another study, which used the AZD6482 inhibitor and aspirin in healthy subjects, had a better antiplatelet effect compared to the combination of clopidogrel and aspirin, with a remarkably lower bleeding risk [69].

Additionally, in this pathway, the protein kinase CK2 is considered an essential regulator for the clustering of IP3 receptors, facilitating the rapid release of Ca^2+^ from internal storage upon platelet activation dependent of GPVI [70]. In a murine model of photochemical thrombosis, the use of CX-4945 (a selective inhibitor of CX2) showed a delay in the induced thrombus formation [71,72]. Another study that used ck2β-/- mice (with a genetic depletion of CK2) showed a significant decrease in the thrombi formation and stabilization without increasing the bleeding times [70].

### 3.3. PKC Signaling Pathway

The protein kinase C (PKC) signaling pathway regulates the platelet response in thrombus formation, where the proteins PKCε and PKCδ play a crucial role in this process [73,74,75]. In the activation of GPVI with collagen, the protein PKCε promotes platelet spreading, secretion, and aggregation [76]. On the other hand, PKC-θ also helps to support adhesion and filopodial generation, but does not affect GPVI-stimulated aggregation or secretion. In contrast, the lack of both isoforms in a murine model showed a significant decrease in aggregation induced by collagen, with a considerable increase in a tail bleeding study [76]. Moreover, studies show that PKC-θ is activated by GPVI agonists; observing a knockout PKC-θ murine model (model of thrombosis induced with FeCl_3_) led to a compromised hemostasis, prolonged bleeding time, an unstable formation of thrombi, and extended arterial occlusion [77,78].

Concerning PKCδ, the platelet activation through GPVI was associated with an increased platelet function. In this process, PKCδ is phosphorylated by Lyn and SHIP-1, and enhances platelet dense granule secretion [79,80].

Other proteins with a vital role in the PKC pathway mediated by GPVI are phospholipase A2 (PLA2) and the adapter protein SLP-76. The activation of the protein phospholipase A2 (especially in isoform PLA2α) is an essential step for the generation of thromboxane A2 (TXA2) stimulated by GPVI [81]. In murine models lacking cPLA2α, the authors observed efficient protection from thromboembolism, however, with a remarkably increased bleeding time [82,83]. About the SLP-76 protein, it has been described that this adapter protein plays an essential role in platelet activation through GPVI, both in the aggregation, shape change, and granule release [84,85]. It is known that the normal expression of SLP-76 is necessary for normal hemostasis [85]. However, a clean GPVI/FcRγ/SLP-76 signal transduction pathway is not required for the platelet activation induced by collagen. Still, it is necessary for maximal response to costimulation with thrombin plus collagen [84]. In a murine knockout model of SLP-76, the authors observed a higher risk of bleeding with a decreased perinatal survival, which could be reversed by the reconstitution of the protein SLP-76 into the bone marrow cells of mice without SLP-76 [85]. Recently studies have pointed out that pyruvate dehydrogenase kinases (PDKs) play a role in the platelet activation through GPVI, mediated by the PKC signaling pathway [86,87,88,89]. A study that used a potent inhibitor of PDK called dichloroacetic acid (DCA) (600 mg/Kg body weight) in mice showed less of a risk of thrombosis, the inhibition of the static adhesion of platelets to collagen, an average bleeding time, and the impaired phosphorylation of tyrosine on Syk and PLCγ2 [87,88]. However, other studies showed that the administration of DCA (200 mg/kg, intraperitoneal) was associated with prolonged bleeding times, as well as an increase in the amount of bleeding compared to that of vehicle-treated animals [88,89]. Moreover, another study that used a murine model of lacking PDK1 showed a protective effect against ischemic stroke and arterial thrombotic occlusion in vivo, a significant increase in the survival time, and an increase in the risk of bleeding [90].

## 4. Advances in GPVI Modulation by Antibodies and Inhibitory Proteins

**Figure 1:**
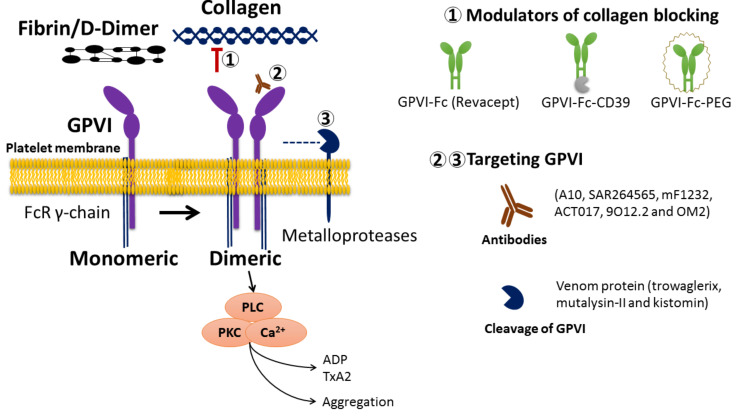
Antiplatelet activity through differential modulation of glycoprotein VI and its collagen binding. The figure shows the main compounds regulating GPVI activity by blocking collagen-GPVI binding (GPVI-FC, GPVI-Fc-CD39, and GPVI-FC-PEG) or direct action on GPVI (antibodies and cleavage). Numbers 1, 2 and 3 indicate each agent’s potential sites of action.

Evidence shows that GPVI inhibition in patients and murine models is related to a significant decrease in thrombus formation induced by collagen without changes in the risk of bleeding [27]. In this way, studying the blocking or antibody-mediated depletion of GPVI becomes a promising pharmacological approach in searching for an effective and safe antiplatelet therapy [91,92,93,94]. On the other hand, the cleavage of GPVI induced by antibodies has been demonstrated to have protective effects against thrombosis in both mice and humans [95]. The different approaches for platelet inhibition through GPVI modulation using antibodies or the cleavage process are described below (Figure 1) [96].

### 4.1. GPVI Antagonists

The use of antibodies to target GPVI in platelets is a promising therapeutic strategy for inhibiting platelet aggregation [94]. Current evidence shows that dimeric anti-GPVI antibodies induce the shedding of GPVI; meanwhile, monomeric anti-GPVI compounds are needed for inhibition without cleavage [94]. The above fact reflects myocardial infarction or ischemic stroke in patients mediated by the exposure of subendothelial collagen after the rupture of atherosclerotic plaques when using antibodies against GPVI, which represents a promising therapy due to the effective prevention of platelet adhesion and thrombus formation within the injured area in the artery [94,97].

Among the currently reported anti-GPVI antibodies with potential therapeutic effects, antibodies based on the Fab fragment represent one of the main strategies. Qian and collaborators developed one of the first studies to search for GPVI antibodies based on Fab fragments. In this study, the authors describe a set of five clones of GPVI-neutralizing human antibodies derived from a combinatorial phage display library of single-chain antibodies; the antibody A10 (150 µg/mL) was the only one capable of inhibiting the binding of GPVI to convulxin, inhibiting collagen-induced aggregation in vitro (collagen 2 µg/mL) [98]. Similarly, Al-Tamimi and collaborators designed an antibody fragment (12A5) with the ability to induce the ectodomain shedding of human GPVI dependent on the metalloproteinase “disintegrin and metalloproteinase” (ADAM) family (with a prominent role of ADAM10), inhibiting the platelet aggregation in vitro (at concentrations of 5, 10, and 20 µg/mL) [99,100]. Following the study of Fab fragments targeting GPVI, Li et al. reported an OM4 Fab antibody capable of inhibiting platelet aggregation induced by collagen in vitro and inhibiting thrombosis in vivo in rat models without increasing the bleeding time (at doses of 20 mg/kg); controversially, these doses induced acute thrombocytopenia [101]. In this way, Matsumoto and collaborators confirmed these findings in cynomolgus monkeys, observing that the OM2 antibody at a concentration of 0.4 mg/kg presented a potent inhibition of aggregation induced by collagen up to six hours after the injection without a significant increase in bleeding time, thrombocytopenia, or the depletion of GPVI [102].

A functional monoclonal antibody-derived Fab fragment against human GPVI was reported by Lecut et al. In this study, the use of the 9O12.2 Fab fragment was able to impair platelet adhesion preventing its formation under in vitro arterial flow conditions (at doses of 50 µg/mL) [103]. Later, Mangin and collaborators confirmed that the injection of 9O12.2 (at doses of 4 mg/kg) into the humanized GPVI mouse model did not prolong the tail bleeding time, but provided significant protection against collagen/adrenaline-induced thromboembolism [104].

Lebozeck et al. reported the production and characterization of a humanized Fab fragment against GPVI, designated as ACT017(glenzocimab), which presented a high capacity to inhibit collagen-induced platelet aggregation ex vivo after injection, specifically (1–8 mg/kg, IC50: 3.2 ± 2.5 µg/mL) to the macaque without inducing thrombocytopenia, GPVI depletion, or bleeding side effects [105]. Ahmed et al. demonstrated through an ex vivo thrombosis model and computational simulation that glenzocimab induced platelet disaggregation under arterial blood flow conditions (at 50 µg/mL). Still, this effect requires GPVI to interact with plasmatic fibrinogen and without thrombin [106]. In 2020, Renaud and collaborators reported a clinical study ACT107 [107]. The authors evaluated the pharmacokinetics and pharmacodynamics of glenzocimab in healthy volunteers, and the ex vivo platelet analysis showed that glenzocimab at doses of 1000 mg (in 6 h IV infusions) reduced platelet aggregation to 20% in 100% of subjects, and 60% in 12 h after dosing [107]. In addition to the above, Voors-Pette and collaborators demonstrated through a clinical trial that an intravenous dose of ACT017 (62.5 to 2000 mg) did not significantly affect the bleeding time in a clinical sense without a change in the number of platelets, the platelet GPVI expression, or plasma levels [108]. Recently, it has been reported that glenzocimab entered a phase II trial in stroke patients (Acute Ischemic Stroke Interventional Study “ACTIMIS) (NCT03803007), which is a significant advance for this therapeutic approach.

### 4.2. Blocking the Binding Site for GPVI on Collagen

Revacept, a protein created with the dimeric fusion of the human Fc fragment and the extracellular domain of GPVI (GPVI-Fc), has shown to induce a significant decrease in thrombus formation after endothelial injury, improve the functional outcome, cerebral infarct size, and edema compared to the control (Fc fragment only) (at doses 1 mg/kg IV) [109]. This protein was evaluated in humans (clinical trial phase I) by Ungerer in the year 2011, demonstrating that revacept is a safe and well-tolerated new antiplatelet compound (inhibitor of platelet aggregation) that is dose-dependent, specific, and does not affect bleeding time [110]. Another study showed that repeated doses of revacept significantly improved endothelial dysfunction and vascular morphology in atherosclerotic rabbits (revacept at 8 mg/kg twice weekly for 4 weeks) [111]. Furthermore, no influence on the bleeding time of revacept alone or in combination with various antiplatelet drugs was found in mice [109,111]. Even when using revacept with other antiplatelet therapies such as ASA and a P2Y12 inhibitor, it was more likely to improve the protection against atherothrombosis without increasing bleeding risk [112]. Specifically, revacept inhibited the plaque-induced platelet aggregation by 53%, and increased platelet inhibition of ASA by 51% to 66%, and ticagrelor by 64% to 80% (at doses of 3–4 mg/kg IV) [112]. Schüpke, Mayer, and collaborators evaluated the use of revacept (80 and 160 mg) in patients undergoing percutaneous coronary intervention (clinical phase II, ISAR-PLASTER trial) (NCT03312855) [113]. Controversially, the results showed that revacept did not reduce myocardial injury in these patients, with a few bleeding events and no significant differences between treatment arms [114]. The high dose of revacept (160 mg) was associated with a small but significant reduction in high-concentration collagen-induced platelet aggregation in these patients, a smaller effect than that observed in animal models [114]. In addition to the above, Gröschel and collaborators evaluated the safety, tolerability, and efficacy of revacept (40 or 120 mg) in patients with carotid artery stenosis, transient ischemic attacks (TIAs), amaurosis fugax, or stroke (clinical phase II, revacept CS02 multicenter trial,) (NCT01645306) [115]. The results showed that revacept at doses of 120 mg reduced the appearance of new ischemic lesions and the risk of adverse health events (stroke, death, myocardial infarction, coronary intervention, and bleeding) [116].

Degen et al. designed a fusion protein by binding GPVI-Fc to ectonucleotidase CD39 (fusion protein GPVI-CD39), adding the ability to stimulate the local adenosine diphosphate (ADP) degradation and showing a significant increase in the inhibition of platelet aggregation and a decrease in the arterial thrombi formation without increase the tail bleeding time in vivo (3 mg/kg), when compared with GPVI-Fc alone [117]. Other cases of a modified GPVI-Fc protein were observed in the study performed by Wufuer and collaborators, when GPVI-Fc was linked to polyethylene glycol (PEG), forming the protein GPVI-Fc-PEG (for a better pharmacological release). This molecule is associated with competitive blocking von Willebrand factor (VWF)–collagen interactions [118]. The administration of GPVI-Fc-PEG (0.18 mg per day) showed an increase in reperfusion and an improvement in the survival following cerebral thrombosis in a murine model, compared with treatment with GPVI alone and without the risk of intracranial bleeding [118].

### 4.3. Proteins Whit a Cleavage Effect on GPVI

Chang and collaborators demonstrated that trowaglerix, a heterodimeric protein derived from a venomous snake (Tropidolaemus wagleri), at low doses could inhibit the platelet aggregation induced by collagen but not by ADP in a murine model (0.8 ng/g), and could induce the loss of GPVI in vitro using human platelets [119]. These effects were associated with trowaglerix’s ability to generate GPVI cleavage by mechanisms dependent on ADAM [119]. In this sense, the polypeptide Troα6 (30 mg/kg) and Troα10 (10 mg/kg), derived from trowaglerix, were able to inhibit the thrombus formation by blocking GPVI in the D1 domain on its lower surface, as well as the D2 domain in the outer surface without increasing the in vivo bleeding time, compared to the aspirin control [120].

Similarly, kistomin, a P–I snake venom metalloproteinase, though its proteolytic activity inhibited the union of collagen and GPVI, it cut near the mucin-like region of GPVI. Kistomin, in a concentration-dependent manner (0.2–12 µM), inhibited the platelet aggregation induced by convulxin but with a slight effect on aggregation induced by collagen [121]. Moreover, it has been reported that kistomin cleaves GPIb and has an additional impact on platelet–VWF interactions [122]. A recent protein derived from snake venom and with a cleavage effect on GPVI was the metalloproteinase called mutalysin-II. This protein blocks collagen-induced platelets through the cleavage of GPIb in washed platelets (in a concentration-dependent manner) without a cleavage effect on VWF, and inhibits the aggregation induced by this same factor [123]. This points to mutalysin-II potentially not being specific, making more studies necessary for the evaluation of the potential therapeutic effects of this protein for clinical development [123].

### 4.4. Chemical Agents with Inhibitory Effect on GPVI

GSK669 is a benzimidazole diamide compound that Glaxo Smith Kline first developed as an anti-inflammatory agent, and was recently reported to have antiplatelet and antithrombotic effects mediated by GPVI [124,125]. This agent is a nucleotide-binding oligomerization domain receptor (NOD2) antagonist, and was able to significantly inhibit platelet aggregation induced by collagen, ATP release, reactive oxygen species (ROS) generation, platelet spreading, and clot retraction (2 mg/kg) compared to the aspirin control and through a mechanism dependent on PKC pathway signaling [124]. Its NOD2 antagonist binds to GPVI and can significantly inhibit platelet aggregation induced by collagen and CRP [124,125]. It also inhibits platelet adhesion to collagen under flow and murine arterial thrombosis and pulmonary embolism in a NOD2-independent manner. A recent study presented the first side-by-side assessment of reported small-molecule GPVI modulators using flow cytometry, aggregometry, and a CRP-XL agonist for the honokiol compounds glaucocalyxin, cinanserin, losartan, and a novel compound developed by Bhunia et al. [126,127]. The results showed that the losartan derivate and the novel compound were the most viable GPVI modulators because of the high specificity and inhibitory potency compared to the other agents [126].

## 5. Conclusions

The search for antithrombotic treatment strategies that do not increase the risk of bleeding and maintain hemostasis is a highly relevant issue in the global panorama of cardiovascular diseases. In this context, the evidence showed that inhibiting GPVI mediated by Fab-based antibodies is an excellent alternative to achieving this goal. Different studies have shown that the inhibition of GPVI with the antibody glenzocimab is a promising therapy due to the crucial advances regarding clinical trials and tests in humans, maintaining the encouraging results observed in animal and in vitro studies. Moreover, the progress of revacept development is similar to that observed concerning glenzocimab, showing promising results, but with some inconsistencies in cardiac patients. However, more extensive clinical trials are necessary to confirm the clinical effects and the absence of side effects.

On the other hand, different authors pointed out that the main advantage of revacept as a therapeutic approach compared to other antithrombotic methods currently under development was its efficacy profile limited to sites of atherosclerotic plaque rupture or after coronary intervention without compromising systemic hemostasis or affecting intrinsic platelet activity; however, results of clinical trials in phase II show that it does not reduce myocardial injury in patients undergoing percutaneous coronary intervention, making it necessary to conduct more studies confirming the clinical effects and safety observed in other clinical trials. Current evidence indicates that GPVI collagen inhibition could be used in potential antithrombotic but not antihemostatic therapy; however, the underlying mechanisms are still unclear. From the authors’ perspectives, this could be due to the fact that the activation of GPVI by collagen may not be essential in primary hemostasis and may be covered by other platelet glycoproteins. However, activation by this platelet agonist at sites of vascular injury might be necessary for thrombus formation.

Finally, this review also presented the basis for different experts and clinicians to discuss future perspectives on GPVI antibodies and molecules targeting GPVI-mediated signaling.

## Data Availability

Not applicable.
